# TPL2-NPM-p53 pathway monitors nucleolar stress

**DOI:** 10.18632/oncoscience.254

**Published:** 2015-10-05

**Authors:** Aristides G. Eliopoulos, Sinisa Volarevic

**Affiliations:** Division of Basic Sciences, University of Crete Medical School, 71003 Heraklion, Crete, Greece

**Keywords:** TPL2, nucleophosmin, nucleolus, p53

Serving as the cellular factory for the biogenesis of ribosomes (the molecular machines responsible for the decoding of mRNAs to proteins), the nucleolus controls a vast array of physiological processes including cell growth and proliferation. It thus comes as no surprise that inherited and acquired abnormalities in ribosome biogenesis can lead to tumorigenesis and that changes in size and number of the nucleoli, which are assumed to reflect the rate of ribosome production, have long been recognized as feature of a large number of tumor types. To safeguard against the potentially tumorigenic effects of deregulated ribosome biogenesis, cells activate the p53 tumor suppressor by re-directing the ribosomal protein (RP) L5/RPL11/5S rRNA pre-ribosomal complex from ribosome biogenesis to HDM2 binding, alleviating its inhibitory effect over p53 [1].

However, it has long been appreciated that upon exposure of cells to distinct genotoxic agents that also perturb nucleolar structure and ribosome biogenesis, the nucleolar proteins nucleophosmin (NPM) and alternative reading frame (ARF) tumor suppressor are engaged to activate p53. Thus, oncogene-induced replication stress and genotoxic insults ensue DNA damage responses that impair the nucleolar interaction of ARF with NPM, leading to the release of ARF to the nucleoplasm where it binds HDM2 and inhibits HDM2-mediated degradation of p53 [2]. NPM is also mobilized to the nucleoplasm, although with somewhat slower kinetics, to associate with free, p53-bound or *de novo* synthesized HDM2 [2]. The ensuing accumulation of p53 is required for cell cycle arrest, senescence or apoptosis of damaged cells. The central role of NPM in these nucleolus-orchestrated responses is further highlighted by the fact that NPM mutations which render it cytoplasmic are associated with genomic instability and the development of hematopoietic malignancies such as acute myeloid leukemia (AML) [2].

**Figure 1:**
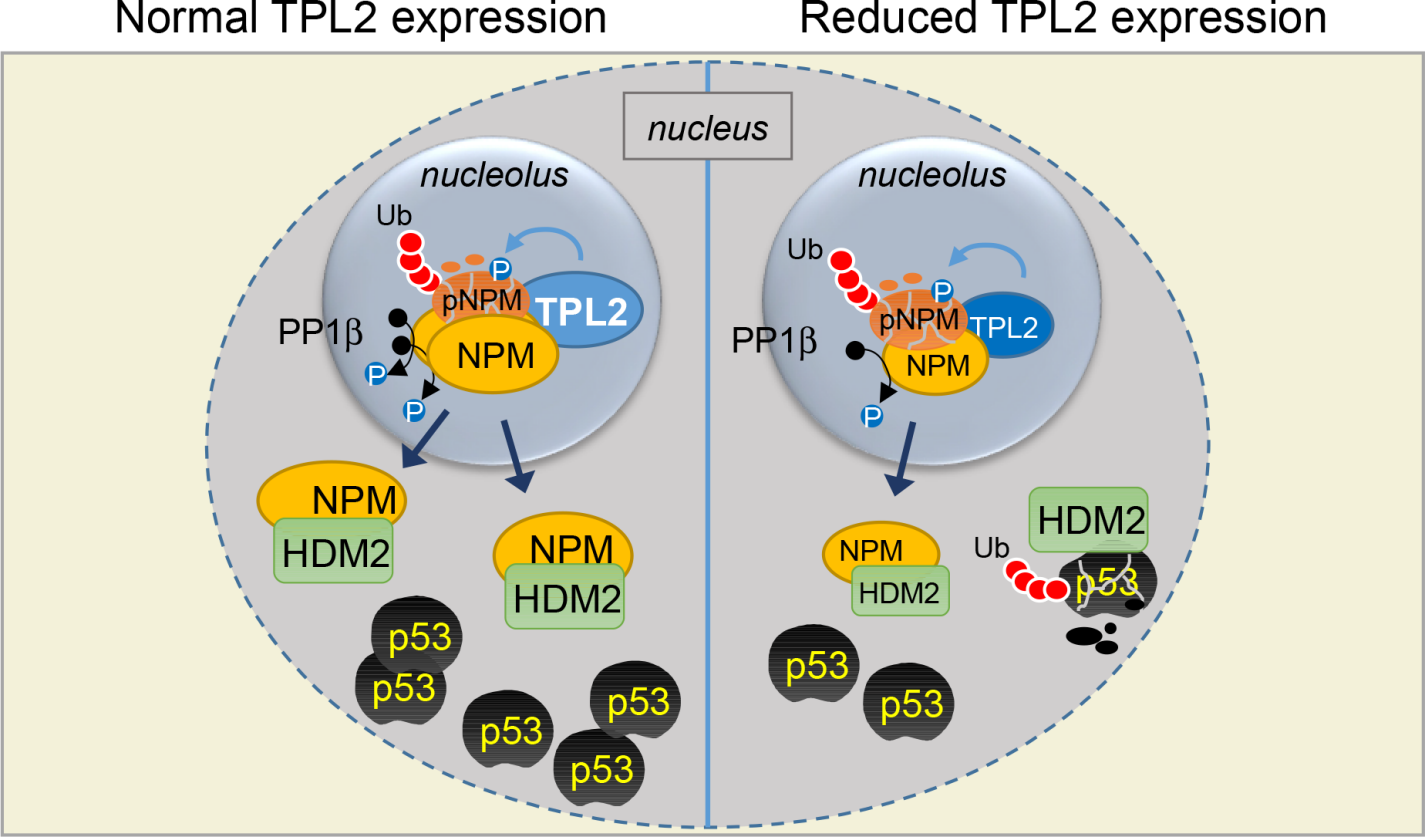
Schematic representation of a novel TPL2/NPM/HDM2/p53 pathway activated upon nucleolar stress [3] TPL2 physiologically functions to phosphorylate a pool of NPM (pNPM) targeting it for ubiquitination and degradation. Following nucleolar stress by UVC, pNPM becomes de-phosphorylated by activated PP1β, stabilizes and translocates to the nucleoplasm where it associates with HDM2 alleviating its effect on p53 degradation (left). The down-regulation of TPL2 expression observed in certain tumor types (right) affects the entire chain of nucleolar and nucleoplasmic events resulting in impaired p53 accumulation (see text for details).

A recent study by Kanellis *et al*. [3] provides novel insight into the intricate management of p53 activation upon “nucleolar stress” by identifying Tumor Progression Locus 2 (TPL2; also known as COT and MAP3K8) as a physical and functional partner of NPM. TPL2 has mostly been appreciated as a cytoplasmic kinase involved in the wiring of pro-inflammatory signal transduction [4]. Kanellis *et al*. have found that in malignant cells and normal fibroblasts a fraction of TPL2 resides in the nucleolus where it associates with and phosphorylates a pool of NPM molecules at Thr^199^. As this phosphorylation event is required for NPM ubiquitination and proteasomal degradation, TPL2 appears to participate in the maintenance of physiological levels of NPM. Upon genotoxic stress this NPM pool becomes de-phosphorylated by activated PP1β [5], stabilizes and translocates to the nucleoplasm where it sequesters HDM2 away from p53 leading to a robust p53 response (Figure 1). Consistent with this novel TPL2 function in p53 activation, several human cancers display reduced expression of TPL2 [4, 6] and recently published genetic evidence in the mouse suggests that this kinase operates in epithelial cells as suppressor of malignant transformation [6, 7].

How do these observations fit in the aforementioned NPM-centered model? The work by Kanellis *et al*. suggests that when TPL2 levels are reduced, the fraction of NPM that becomes phosphorylated diminishes. This in turn results in a limited pool of phosphorylated NPM available for PP1β-mediated de-phosphorylation upon nucleolar stress, causing insufficient mobilization of NPM to the nucleoplasm thereby allowing HDM2 to maintain significant control over p53 (Figure 1). In line with these observations, NPM or TPL2 silencing impairs stress-induced p53 stabilization *in vitro* and *in vivo* [2, 3, 6].

This work hints to an unprecedented duality in TPL2 function that depends on subcellular topology: transduction of pro-inflammatory signals in the cytoplasm and inhibition of tumorigenic pathways in the nucleus. However, a number of outstanding questions remain unanswered. How do these distinct TPL2 functions intertwine to achieve control of processes relevant to tumorigenesis such as cell cycle arrest, apoptosis and senescence? What is the relationship between nucleolar TPL2 and oncogene-induced ARF or the RPL5/RPL11/5S rRNA/HDM2/p53 signaling pathway? Do these pathways sense distinct stress-induced nucleolar lesions and do they synergize in p53 activation and tumor suppression?

An abundance of evidence has shown that inherited abnormalities in ribosome function can lead to tumorigenesis. Diamond Blackfan Anemia (DBA) is a rare inherited bone marrow failure syndrome caused by alterations in several RP genes and it typifies a group of disorders called ribosomopathies that are linked to mutations in genes encoding RPs or other factors involved in ribosome biogenesis [8]. DBA itself predisposes to myelodysplastic syndromes (MDS) which may further progress to AML. As errors in ribosome biosynthesis activate the RPL5/RPL11/5S rRNA/HDM2/p53 signaling pathway, it has been suggested that the selection of cells that loose expression of wild-type p53 or acquire mutations in p53 or other signaling components of the RPL5/RPL11/5S rRNA/HDM2 complex may allow escape from the ribosome biogenesis stress-imposed checkpoint, thereby facilitating tumor progression. As AML cells show approximately 2-fold reduction in TPL2 mRNA levels [4], mutational inactivation of p53 and cytoplasmic localization of NPM [2], it can be postulated that the TPL2/NPM/HDM2/p53 response might also, alone or in synergy with the RPL5/RPL11/5S rRNA/HDM2/p53 signaling pathway, inhibit progression of pre-neoplastic MDS cells to AML, a possibility that warrants further investigations.

The identification of TPL2 as a novel component of the nucleolar stress response may therefore contribute to better understanding of the pathogenesis of human diseases and the pursuit of TPL2 signaling will no doubt continue to provide invaluable insight into the complex biological functions of the nucleolus.
